# Prevalence of Intrathecal Acyclovir Resistant Virus in Herpes Simplex Encephalitis Patients

**DOI:** 10.1371/journal.pone.0155531

**Published:** 2016-05-12

**Authors:** Johanna G. Mitterreiter, Maarten J. Titulaer, Gijsbert P. van Nierop, Jeroen J. A. van Kampen, Georgina I. Aron, Albert D. M. E. Osterhaus, Georges M. G. M. Verjans, Werner J. D. Ouwendijk

**Affiliations:** 1 Research Center for Emerging Infections and Zoonoses, University of Veterinary Medicine Hannover, Hannover, Germany; 2 Department of Viroscience, Erasmus MC, Rotterdam, the Netherlands; 3 Department of Neurology, Erasmus MC, Rotterdam, the Netherlands; Geisel School of Medicine at Dartmouth, UNITED STATES

## Abstract

Herpes simplex encephalitis (HSE) is a life-threatening complication of herpes simplex virus (HSV) infection. Acyclovir (ACV) is the antiviral treatment of choice, but may lead to emergence of ACV-resistant (ACV^R^) HSV due to mutations in the viral *UL23* gene encoding for the ACV-targeted thymidine kinase (TK) protein. Here, we determined the prevalence of intrathecal ACV^R^–associated HSV TK mutations in HSE patients and compared TK genotypes of sequential HSV isolates in paired cerebrospinal fluid (CSF) and blister fluid of mucosal HSV lesions. Clinical samples were obtained from 12 HSE patients, encompassing 4 HSV type 1 (HSV-1) and 8 HSV-2 encephalitis patients. HSV DNA load was determined by real-time PCR and complete HSV TK gene sequences were obtained by nested PCR followed by Sanger sequencing. All HSV-1 HSE patients contained viral TK mutations encompassing 30 unique nucleotide and 13 distinct amino acid mutations. By contrast, a total of 5 unique nucleotide and 4 distinct amino acid changes were detected in 7 of 8 HSV-2 patients. Detected mutations were identified as natural polymorphisms located in non-conserved HSV TK gene regions. ACV therapy did not induce the emergence of ACV^R^-associated HSV TK mutations in consecutive CSF and mucocutaneous samples of 5 individual patients. Phenotypic susceptibility analysis of these mucocutaneous HSV isolates demonstrated ACV-sensitive virus in 2 HSV-1 HSE patients, whereas in two HSV-2 HSE patients ACV^R^ virus was detected in the absence of known ACV^R^-associated TK mutations. In conclusion, we did not detect intrathecal ACV^R^-associated TK mutations in HSV isolates obtained from 12 HSE patients.

## Introduction

Herpes simplex encephalitis (HSE) is a rare life-threatening complication of infection with herpes simplex virus type 1 (HSV-1) and HSV-2. HSE affects 1 in 250,000 to 500,000 persons per year in the USA and comprises about one-fifth of all encephalitis cases in the UK [1,2]. HSV-1 causes 90% of HSE cases in immunocompetent adults, whereas HSV-2-associated HSE is more common in neonates and immunocompromised individuals [2,3]. Prompt diagnosis, based on magnetic resonance imaging of the brain and detection of HSV DNA in cerebrospinal fluid (CSF) by PCR, and preemptive administration of antiviral drugs are pivotal to reduce HSE-induced morbidity and mortality [2–4]. Mortality from HSE is about 70% in untreated patients, which is reduced to 11% to 19% when immediate antiviral therapy is provided [1,5]. Nevertheless, approximately 12% to 60% of treated HSE patients develop neurological sequelae [1,5].

Treatment of choice is intravenous administration of the antiviral agent acyclovir (ACV), a highly selective drug for human alphaherpesviruses with limited side effects [6,7]. ACV is a prodrug converted by the viral thymidine kinase (TK) protein to ACV-monophosphate, which is subsequently converted by cellular kinases to the active compound ACV-triphosphate that inhibits HSV DNA replication [8]. ACV resistant (ACV^R^) HSV is found in less than 1% of immunocompetent, but more frequently in immunocompromised individuals (4% to 30%) and in infected immunoprivileged organs including the cornea [8–11]. Over 95% of ACV^R^ HSV are caused by mutations in the viral *UL23* gene encoding the ACV-targeted TK protein, while mutations in the viral *UL30* DNA polymerase gene account for the remaining 5% [12]. Few studies reported on ACV treatment refractory HSE cases due to infection with ACV^R^ HSV, but the overall prevalence of intrathecal ACV^R^ HSV in a clinically unbiased cohort of patients with either HSV-1 or HSV-2-induced HSE has not been described [13–16]. Furthermore, the highly polymorphic nature of the HSV-1 *UL23* TK gene, and to a lesser account its HSV-2 counterpart, facilitates genetic differentiation of HSV isolates [10,17,18].

The aim of this study was twofold. First, to determine the prevalence of intrathecal ACV^R^ HSV in a cohort of 4 HSV-1 and 8 HSV-2 HSE patients. Second, to compare the HSV TK genotype variation of sequential CSF and/or blister fluid samples (BLS) obtained from the same patient during the course of disease.

## Materials and Methods

### Clinical specimens

Surplus CSF samples of suspected HSE patients, obtained for diagnostic purposes at the Erasmus Medical Center (Rotterdam, the Netherlands), were collected between 2001 to 2012. A total of 15 CSF samples from 12 HSE patients were included: HSV-1 (n = 4) and HSV-2 (n = 8) (Table 1). Additionally, paired BLS from mucocutaneous lesions of two HSV-1 (patients #3 and #4) and two HSV-2 HSE patients (patients #10 and #11) were obtained. According to our institutional “Opt-Out” system, which is defined by the National "Code of Good Conduct" (Dutch: Code Goed Gebruik, May 2011) [19], surplus clinical materials are made available for research purposes if the patient has not objected. Specifically, patients/donors or caretakers that object are registered and their specimens are excluded from future research. Accordingly, the current study used only surplus clinical samples from HSE patients that did not object use of their surplus clinical samples for future research purposes. The study protocol was approved by the medical ethical committee of the Erasmus MC (MEC-2012-226) and informed consent was waived. The study was performed according to the tenets of the Helsinki declaration.

**Table 1:** Demographics and clinical characteristics of herpes simplex encephalitis patients.

Patient	Virus^a^	Age, Sex^b^	Immune status^c^	Time onset–first CSF^d^	Time onset–AV therapy^e^	AV therapy (duration in days)^f^	Follow-up (months)	Clinical outcome at end of follow-up^g^
1	HSV-1	51, F	-	11 months	None	None	142	Moderate disability
2	HSV-1	35, M	+	4 days	4 days	ACV iv (14); GCV iv (28)	18	Complete recovery
3	HSV-1	64, F	+	3 days	5 days	ACV iv (14); ACV oral (7)	1	Complete recovery
4	HSV-1	47, M	-	14 days	10 days	ACV iv (±14–21)	114	Severe disability
5	HSV-2	59, M	-	21 days	22 days	ACV iv (10)	24	Complete recovery
6	HSV-2	36, F	+	4 days	4 days	ACV iv (±14–21)	Unknown	Unknown
7	HSV-2	19, F	+	1 day	3 days	ValGCV oral (12 days)	7	Complete recovery
8	HSV-2	49, F	+	1 day	1 day	ValACV oral (14)	38	Moderate disability
9	HSV-2	61, M	-	10 days	10 days	ACV iv (28); ValACV oral (21)	18	Good recovery
10	HSV-2	28, M	-	5 days	5 days	ACV iv (14)	108	Complete recovery
11	HSV-2	35, M	-	± 2 months	14 days	ACV iv (2); none (32); ValACV oral (39)	3	Good recovery
12	HSV-2	61, M	-	8 days	3 days	ACV iv (19)	3	Good recovery

^a^ Causative virus.

^b^ Age in years. F: female; M: male.

^c^ +: immunocompetent;—: immunocompromised.

^d^ Time interval between the onset of clinical symptoms and time of (first) cerebrospinal fluid (CSF) sample.

^e^ Time interval between the onset of clinical symptoms and the onset of antiviral (AV) therapy.

^f^ Successive antiviral therapies and routes of administration are indicated. ACV: acyclovir; GCV: ganciclovir; ValACV: valacyclovir; ValGCV: valganciclovir; iv: intravenous.

^g^ Complete recovery: no adverse events; other outcome criteria classified according to Glasgow outcome scale [22].

### DNA extraction and quantitative real-time PCR

DNA was extracted from CSF and BLS samples using the QIAamp DNA Mini Kit (Qiagen) according to manufacturer’s instructions. Quantitative PCR (qPCR) for HSV was performed using an ABI Prism 7500 Real-Time PCR System and TaqMan Universal PCR Master Mix (Life Technologies) with primers and probes specific for HSV-1 and HSV-2 as described before [20]. Electron microscopy quantified high-titer HSV stocks (Advanced Biotechnologies Incorporated) were used for standardization of qPCR assays [20]. The lower limit of detection was 50 HSV genome equivalent copies per mL.

### HSV TK gene sequencing

The entire HSV-1 and HSV-2 TK genes (both 1,131 bp long) were amplified from DNA extracted from clinical specimens by nested PCR using PfuUltra II Fusion HS DNA Polymerase (Stratagene), 5% dimethyl sulfoxide (Sigma-Aldrich) and the following primers at the indicated concentrations: HSV-1 external forward 5’-GCGGTCCCAGGTCCACTTC-3’ (1.2 μM), HSV-1 external reverse 5’-cacccgtgcgttttattctgtc-3’ (1.2 μM), HSV-1 nested forward 5’-atcttggtggcgtgaaactcc-3’ (0.4 μM), HSV-1 nested reverse 5’-ggttccttccggtattgtctcc-3’ (0.4 μM), HSV-2 external forward 5’-gtcagcagcgttccacaaatcc-3’ (0.6 μM), HSV-2 external reverse 5’-ggggtggggtgagggtaaaag-3’ (0.6 μM), HSV-2 nested forward 5’-cgttgaactcccgcacctctc-3’ (0.4 μM) and HSV-2 nested reverse 5’-cccccgcgcttatggacac-3’ (0.4 μM) (all from Eurogentec) [10]. First round amplification conditions included denaturation for 2 min at 95°C, followed by 40 cycles 30 s at 95°C, 25 s at 62°C, 40 s at 72°C and a final elongation of 3 min at 72°C. Nested PCR was performed, using 1 μl of the first PCR reaction, for 35 cycles using identical conditions. The resulting TK amplicons were purified from agarose gel using the MiniElute Gel Extraction Kit (Qiagen) and sequenced on the ABI Prism 3130 XL Genetic Analyzer with the BigDye v3.1 Cycle Sequencing Kit (both Applied Biosciences), the above-mentioned nested primers, and the following additional internal primers at the indicated concentrations: HSV-1 internal forward 5'-CGCCCAGATAACAATGGGC-3' (1 μM), HSV-1 internal reverse 5'-CCCATAAACGCGGCGAATCG-3' (1 μM), HSV-2 internal forward 5'-accaggttcgtgccgggcgcggtc-3' (1 μM) and HSV-2 internal reverse 5'-tatcgcctccctgctgtgctaccc-3'(1 μM), as described [10,21]. TK sequences were aligned to consensus TK sequences of the HSV-1 reference strain 17 (GenBank accession number: JN555585.1) and HSV-2 reference strain HG52 (NCBI accession number: NC_001798.1) using Lasergene 10.1 (DNASTAR). Obtained HSV-1 and HSV-2 TK sequences were deposited in GenBank under the accession numbers KT266758 –KT266777.

### Phenotypic ACV susceptibility of HSV isolates

Phenotypic ACV susceptibilities of herpes simplex virus isolates were determined by plaque reduction assay according to the Clinical and Laboratory Standards Institute’s guidelines. In brief, confluent monolayers of Vero cells cultured in 12-well plates were infected with 50–100 plaque forming units HSV per well for 75 min at 36.5°C in a CO_2_ incubator. Inoculum was removed and EMEM containing 0.8% agarose (both Lonza) and 0–64 mg/mL ACV (GlaxoSmithKline) was added. Plates were incubated at 36.5°C with 5% CO_2_ for 72 hours, fixed using 10% formalin and stained with crystal violet. Plaques were counted using a light microscope. HSV reference strains HSV-1 F and HSV-2 HG52 were included as ACV-sensitive controls. GraphPad Prism 5 was used to calculate the half maximum (50%) inhibitory concentrations (IC_50_) of ACV from the dose response curves using the log(inhibitor) versus normalized response with a variable Hill slope. Experiments were performed in duplicate and three independent experiments were performed. HSV isolates were considered ACV^R^ if the mean IC_50_ was equal or greater than five times the IC_50_ value of the sensitive control HSV-1 or HSV-2 strains.

## Results

### Demographics and clinical characteristics of HSE patients

Fifteen CSF samples were obtained from 12 HSE patients comprising 4 HSV-1 and 8 HSV-2 HSE cases (Table 1). Five of 12 (42%) of patients were female, the overall median age at time of the first CSF sample was 48 years (range: 19–64 years) and 7 of 12 patients (58%) were immunocompromised. Age, gender and immune status distribution were comparable between HSV-1 and HSV-2 HSE patients. Median time interval between onset of disease and first CSF sample was 6.5 days (range: 1 day– 11 months). Eleven of 12 patients (92%) received antiviral therapy, starting at a median time interval of 5 days (range: 1–22 days) after onset of disease symptoms. Patient #1 did not receive antiviral therapy because she was initially suspected of and treated for tuberculous meningoencephalitis; HSV-1 DNA was detected in CSF at 11 months after onset of neurological disease. The median follow-up was 18 months (range: 1 month– 11 years); one patient was lost to follow-up. Five of 11 patients (45%) showed complete recovery, 3 of 11 patients (27%) showed good recovery, 2 of 11 (18%) patients showed moderate disabilities and 1 patient developed severe disabilities according to the Glasgow outcome scale [22].

### Prevalence of ACV^R^ HSV in CSF from HSE patients

To determine the prevalence of intrathecal ACV^R^ HSV in the cohort of clinically unbiased HSV-1 (n = 4) and HSV-2 (n = 8) HSE patients, DNA was isolated from surplus CSF and subsequently used to amplify the complete viral TK gene by nested PCR followed by Sanger sequencing. Median HSV-1 and HSV-2 DNA loads in the primary CSF samples of the HSE patients were 3.7 x 10^4^ copies/mL (range <50–1.3 x 10^6^ copies/mL) and 1.8 x 10^4^ copies/mL (range 5.0x10^2^–2.2x10^5^ copies/mL), respectively (Table 2). HSV-1 and HSV-2 TK sequences were obtained from all CSF samples analyzed, irrespective of viral DNA load. Among HSV-1 HSE patients, all CSF samples had viral TK mutations comprising 30 unique nucleotide and 13 distinct amino acid substitutions compared to HSV-1 reference strain 17 (Table 2). By contrast, TK sequences in 7 of 8 (88%) CSF samples from HSV-2 HSE patients contained mutations, encompassing only 5 unique nucleotide and 4 distinct amino acid substitutions compared to HSV-2 reference strain HG52.

**Table 2:** Viral thymidine kinase polymorphisms in clinical samples obtained from herpes simplex encephalitis patients.

						Herpes simplex virus (HSV) thymidine kinase (TK) gene	
			Time interval				
Patient	Virus^a^	Clinical Sample^b^	First CSF- sample^c^	Onset AV therapy—sample^d^	Viral load (gec/mL)^e^	Nucleotide polymorphisms^f^	Amino acid polymorphisms^g^
1	HSV-1	CSF	NA	NA	<5.0 x10^1^	A106G, *C171T*, G266A, *T271C*, *T717C*, G793A, *C892T*, *T933C* and *C1056T*	K36E, R89Q and A265T
2	HSV-1	CSF	NA	0	7.9 x 10^3^	A68G, *G102A*, A106G, *C171T*, G266A, *T271C*, *C513T*, **C689T**, T717C, G719A, *G793A*, *T933C* and *A1065C*	N23S, K36E, R89Q, **A230V**, G240E and A265T
3	HSV-1	CSF1	NA	-2	1.3 x 10^6^	A106G, *C171T*, G266A, *T271C*, *T717C*, G793A, *C892T*, *T933C* and *C1056T*	K36E, R89Q and A265T
		BLS	2	0	1.4 x 10^4^	Identical to CSF1	Identical to CSF1
		CSF2	3	1	3.5 x 10^5^	Identical to CSF1	Identical to CSF1
		CSF3	10	8	4.4 x 10^3^	Identical to CSF1	Identical to CSF1
4	HSV-1	BLS1	-4	0	8.0 x 10^6^	*T16G*, *A24G*, A68G, A106G, **G122A**, *C171T*, *T271C*, *A528G*, C575T, *C672T*, *T717C*, *G723A*, G751T, G793A, G799T, C802A, C858A, *T915C*, *T933C*, *C1053T* and A1126C	N23S, K36E, **R41H**, A192V, G251C, A265T, V267L, P268T, D286E and N376H
		BLS2	-1	3	3.8 x 10^7^	Identical to BLS1	Identical to BLS1
		CSF	NA	4	6.6 x 10^4^	Identical to BLS1	Identical to BLS1
5	HSV-2	CSF	NA	-1	2.2 x 10^5^	G116A	G39E
6	HSV-2	CSF	NA	0	1.2 x 10^4^	G116A, A232G, G420T and *C852T*	G39E, N78D and L140F
7	HSV-2	CSF	NA	-2	2.3 x 10^4^	None	None
8	HSV-2	CSF	NA	0	1.0 x 10^3^	G116A	G39E
9	HSV-2	CSF1	NA	0	7.2 x 10^4^	G116A	G39E
		CSF2	9	9	3.9 x 10^3^	Identical to CSF1	Identical to CSF1
10	HSV-2	CSF	NA	0	2.1 x 10^5^	G116A	G39E
		BLS	132	132	1.3 x 10^5^	Identical to CSF	Identical to CSF
11	HSV-2	BLS	-6	34	1.9 x 10^7^	G116A and A752G	G39E and Q251R
		CSF	NA	40	5.9 x 10^3^	Identical to BLS	Identical to BLS
12	HSV-2	CSF	NA	5	4.9 x 10^2^	G116A	G39E

^a^ Causative virus.

^b^ Sample type. CSF: cerebrospinal fluid; BLS: blister swab.

^c^ Time interval (days) between the time point of first CSF sample and acquisition of the indicated sample. NA, not applicable.

^d^ Time interval (days) between the start of antiviral (AV) treatment and the time of sampling. NA, not applicable. Values <0: sample obtained prior to onset AV therapy; Value = 0: AV therapy started on the day of sampling; Values >0: sample obtained after onset of AV therapy.

^e^ HSV genome equivalent copies per mL (gec/mL).

^f^ TK DNA sequences were compared to reference HSV-1 TK (strain 17; GenBank accession number JN555585.1) and HSV-2 TK (strain HG52; NCBI accession number NC_001798.1) sequences. Regular font: natural non-synonymous polymorphisms; *italics*: natural synonymous polymorphisms; bold: new mutation; bold and underlined: mutation with unclear significance for ACV resistance.

^g^ Predicted TK protein sequences were compared to to reference HSV-1 TK (strain 17; GenBank accession number JN555585.1) and HSV-2 TK (strain HG52; NCBI accession number NC_001798.1) sequences. Regular font: natural polymorphisms; bold: new mutation; bold and underlined: mutation with unclear significance for ACV resistance.

**Fig 1:**
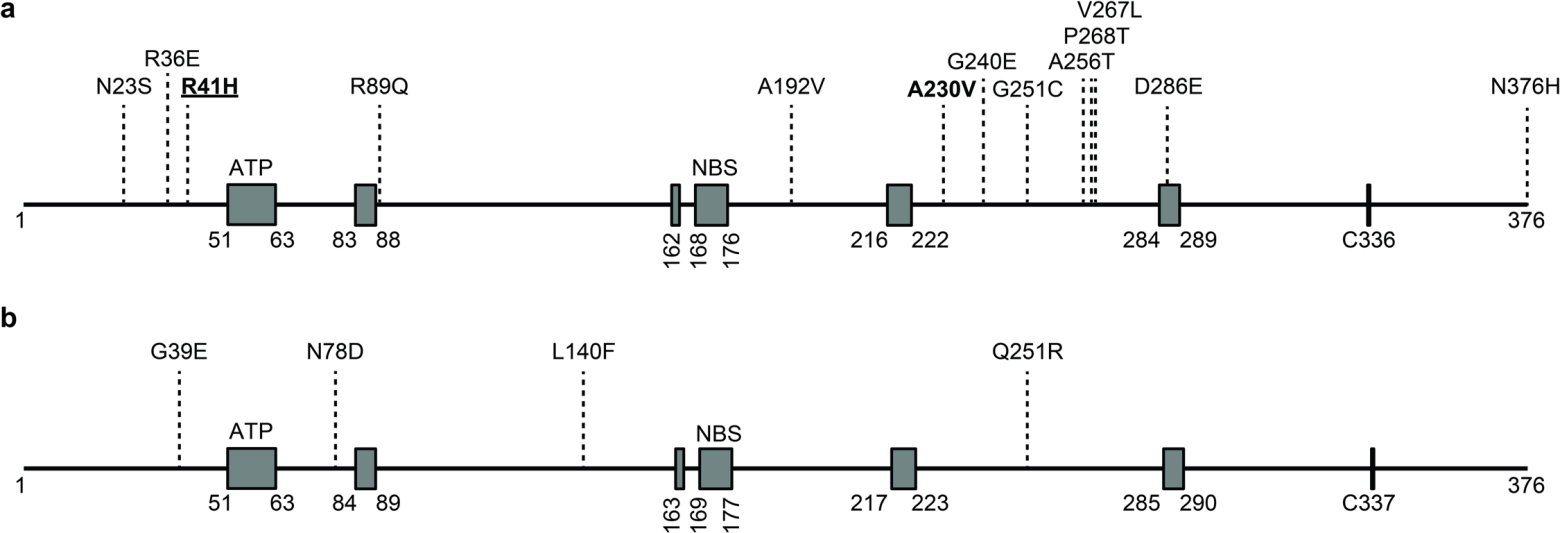
Location of identified amino acid substitutions in HSV thymidine kinase. Schematic representation of all identified amino acid substitutions in the thymidine kinase (TK) proteins of intrathecal HSV-1 (**A**) and HSV-2 (**B**) isolates obtained from herpes simplex encephalitis patients. Note that except for the D286E mutation in HSV-1 all amino acid substitutions are located outside of the functional domains of TK. Grey boxes depict highly conserved and functional domains, i.e. ATP-binding site (ATP), nucleotide binding sites (NBS) and a cysteine residue at position 336 and 337 for HSV-1 and HSV-2, respectively [31, 32]. TK amino acid locations are indicated according to HSV-1 reference strain 17 (GenBank accession number: JN555585.1) and HSV-2 reference strain HG52 (NCBI accession number: NC_001798.1). Regular font: natural polymorphisms; **bold**: new mutation; **bold and underlined**: mutation with unclear significance for ACV resistance.

To predict the impact of the detected mutations on TK function, we determined their location with respect to highly conserved and functional domains of HSV-1 and HSV-2 TK (Fig 1). Except for the D286E mutation in HSV-1 TK protein of patient #4, all amino acid substitutions were located in non-conserved TK domains. All HSV-2 TK and 11 of 13 (85%) HSV-1 TK amino acid substitutions were previously defined as natural polymorphisms resulting in an ACV sensitive (ACV^S^) HSV phenotype [10–12, 23–27]. The HSV-1 TK isolate from patient #4 contained the R41H mutation, previously described as natural polymorphism but also as ACV^R^-associated mutation [10,12,28–30]. One new non-synonymous mutation (C689T) was identified in CSF of HSV-1 HSE patient #2 resulting in the exchange of the nonpolar neutral amino acid alanine at position 230 with a similar nonpolar neutral amino acid valine (Table 2). Collectively, the data indicate that the prevalence of intrathecal ACV^R^-associated TK mutations in HSV-1 and HSV-2 in HSE patients is low.

### Genetic characterization of HSV TK in sequential virus isolates of individual HSE patients

Besides the causative role of TK mutations in the viral ACV susceptibility phenotype, the variability of the TK provides insight into the clonal composition of an HSV-1 isolate [10,17,18]. From 4 HSE patients multiple CSF, and/or BLS from oral HSV-1 or anal HSV-2 mucosal lesions were obtained during the same HSE episode (patients #3, #4, #9, and #11). Additionally, we examined one HSV-2 HSE patient (patient #10) who experienced a genital herpes episode about 4.5 months after HSE (Table 2). To compare TK genotype variation in HSV isolates from concurrent or consecutive HSE and mucosal HSV infections, we sequenced the TK genes of HSV DNA obtained from multiple BLS and CSF samples of the same HSE patient (Table 2). TK sequences were identical for all sequential isolates of individual patients, including those from distinct anatomical HSV infection sites, suggesting potential involvement of the same HSV strain per time and anatomical site.

Because HSV isolates consist of mixtures of ACV^S^ and ACV^R^ variants [8–11], ACV therapy may select for ACV^R^ variants; particularly in immunocompromised individuals and in immunoprivileged organs such as the eye and brain [8–11]. To determine the effect of ACV treatment on the appearance of ACV^R^ HSV in HSE patients, we analyzed viral TK sequences in 3 consecutive CSF samples obtained from two HSE patients during ACV treatment (patients #3 and #9; Table 2). Whereas ACV treatment reduced intrathecal HSV DNA loads, emergence of ACV^R^-associated TK mutations in intrathecal HSV could not be detected (Tables 1 and 2). Likewise, we did not detect ACV^R^-associated mutations in HSV TK of CSF samples obtained at 4 and 40 days after onset of ACV therapy (patients #4 and #11, respectively). Thus, short-term ACV therapy did not select for detectable intrathecal ACV^R^-associated mutations in HSV TK in these 4 HSE patients.

### Phenotypic ACV susceptibility of HSV isolates from HSE patients

Next we aimed to phenotypically characterize the ACV susceptibility of HSE isolates from HSE patients. HSV was isolated from BLS of oral HSV-1 and anal HSV-2 mucosal lesions (Tables 2 and 3), but could not be recovered from CSF due to the low viral loads and/or limited volume of residual sample. These findings are in accordance with the low success rate in recovering culturable HSV from CSF samples (<4%) obtained from HSE patients [33, 34]. As outlined above, TK genotypes were identical in CSF and BLS samples from patients #3, #4, #10 and #11 (Table 2). HSV-1 isolates from patients #3 and #4, the latter containing the disputed R41H TK mutation, were not ACV^R^ (Table 3). By contrast, HSV-2 BLS isolates from patient #10 and #11 obtained at 132 and 34 days after onset of ACV therapy were found to be ACV^R^ in the absence of ACV^R^-associated TK mutations (Table 3 and Fig 1), suggesting involvement of additional mutations in the viral DNA polymerase gene.

**Table 3:** Acyclovir susceptibility of HSV isolates obtained from mucocutaneous samples of herpes simplex encephalitis patients.

Patient	Virus^a^	Sample^b^	Amino acid polymorphisms^c^	Mean [± SD] IC_50_ of ACV (μM)^d^	ACV susceptibility^e^
3	HSV-1	BLS	K36E, R89Q and A265T	0.34 [± 0.05]	Sensitive
4	HSV-1	BLS1	N23S, K36E, **R41H**, A192V, G251C, A265T, V267L, P268T, D286E and N376H	3.50 [± 0.55]	Sensitive
		BLS2	Identical to BLS1	2.85^f^	Sensitive
10	HSV-2	BLS	G39E	14.27 [± 1.52]	Resistant
11	HSV-2	BLS	G39E and Q251R	20.17 [± 6.73]	Resistant

^a^ Causative virus.

^b^ Sample type. BLS: blister swab.

^c^ Predicted TK protein sequences were compared to to reference HSV-1 TK (strain 17; GenBank accession number JN555585.1) and HSV-2 TK (strain HG52; NCBI accession number NC_001798.1) sequences. Regular font: natural polymorphisms; bold: new mutation; bold and underlined: mutation with unclear significance for ACV resistance.

^d^ Mean half maximum (50%) inhibitory concentration (IC_50_) value of ACV ± standard deviation (SD) for the indicated isolates (n = 3 experiments). Mean IC_50_ value for the sensitive control strains HSV-1 F and HSV-2 HG52 were 1.11 μM and 1.93 μM, respectively.

^e^ Isolates were designated as “Sensitive” or “Resistant” based on the ACV IC_50_ values. Cut-off value for ACV resistance was defined as ≥5 times the mean IC_50_ value of HSV-1 F strain (5.55 μM) and HSV-2 HG52 strain (9.65 μM).

^f^ IC_50_ value represents mean of duplicate wells from a single experiment.

## Discussion

Preemptive intravenous ACV therapy has reduced morbidity and mortality in clinically suspected HSE patients [5–7]. Although ACV therapy refractory HSE case reports with intrathecal ACV^R^ HSV have been described [13–16], the prevalence of intrathecal ACV^R^ HSV-1 and HSV-2 in HSE patients is unknown. The current study showed that intrathecal ACV^R^-associated HSV TK mutations are uncommon in a cohort of clinically unbiased HSV-1 (n = 4) and HSV-2 (n = 8) HSE patients and do not appear to develop intrathecally.

Synonymous and non-synonymous mutations were detected in the HSV TK gene from 11 of 12 (92%) HSE patients, most of which were located outside the conserved regions of TK and have been described as natural polymorphisms [10–12, 23–27]. Patient #4 contained the D286E mutation located within the C-terminal conserved domain of HSV-1 TK (amino acid 284–289). This mutation was previously reported as natural polymorphism [10, 12, 23, 25, 26], which did not confer phenotypic ACV^R^ in the BLS HSV-1 isolate of patient #4, possibly because this mutation replaces the negatively charged amino acid aspartic acid with a similarly charged glutamic acid. CSF and BLS of patient #4 contained an HSV-1 isolate harboring the R41H TK mutation. This mutation has been identified in an intrathecal ACV^R^ HSV-1 isolate from an HSE patient refractory to ACV therapy [16] and we have previously shown that a recombinant HSV-1 TK protein containing R41H did not convert ACV to ACV-monophosphate *in vitro* [30]. By contrast, the R41H TK in the HSV-1 isolate from patient #4 did not confer ACV resistance (Table 3) corroborating previous reports describing this mutation as natural polymorphism [12,26,28]. The HSV-1 isolate from patient #2 contained a new mutation (A230V) located in the non-conserved region of HSV-1 TK (Fig 1) that is unlikely to affect TK function, although this needs to be experimentally verified in functional TK assays [18,35,36].

Consistent with previous studies our data indicate a higher prevalence of polymorphisms in the TK gene of HSV-1 compared to HSV-2 [12,21,37]. The genetic variability of the HSV-2 DNA polymerase gene exceeds that of the TK gene [38], suggesting a more prominent role of DNA polymerase mutations in the acquisition of ACV^R^ in HSV-2 isolates. Notably, 2 HSV-2 BLS isolates obtained at 34 and 132 days after onset of ACV therapy from 2 immunocompromised patients (#10 and #11) were found to be phenotypically ACV^R^ in the absence of known ACV^R^-associated TK mutations, suggestive of accompanying mutations in the viral DNA polymerase gene [12]. Alternatively, the isolate’s ACV^R^ phenotype may be caused by G39E (patient #10) or G39E & Q251R (patient #11) HSV-2 TK polymorphisms in combination with mutations in genes encoding additional components of the viral DNA replication machinery:*UL5*, *UL8*, *UL9*, *UL29*, *UL42* or *UL52* [38, 39]. Interestingly, HSV ACV^R^ did not affect clinical outcome in these 2 patients (Table 1). Overall, the data suggests that combined sequence analysis of viral TK and DNA polymerase genes is needed to determine the prevalence of ACV^R^ in CSF samples of HSE patients in future studies.

Previous studies showed that ACV therapy was not associated with an increased risk for the emergence of ACV^R^ HSV-1 in immunocompetent patients [40,41], supporting the currently recommended 14 to 21 days of intravenous ACV treatment of HSE patients [5,42]. The current study showed that HSV TK polymorphisms were present in CSF of 9 patients prior to the onset of ACV therapy. Furthermore, short-term ACV therapy (median: 8 days; range: 1–40 days) did not induce the emergence of ACV^R^-associated TK mutations in HSV isolates obtained from CSF samples of 4 HSE patients (Table 2). However, HSV clinical isolates are quasispecies composed of genetically related ACV^S^ and ACV^R^ variants [10,21,43]. The relative abundance of ACV^S^ and ACV^R^ HSV determines the isolate’s susceptibility to ACV [10,43,44]. Due to limited quantities of HSV DNA recovered from rare CSF samples, we applied pool sequencing to determine the TK sequence of the major HSV isolate present in CSF or mucocutaneous samples. Whereas this reflects the responsiveness of HSE patients to ACV therapy, deep sequencing of HSV TK in future studies should provide more insight into the anticipated subtle emergence of intrathecal ACV^R^ viruses in response to short-term ACV treatment of HSE patients.

In addition to its role in ACV^R^, the genetic variability of the viral *UL23* TK gene facilitates discrimination between HSV-1 strains [10,17,18,30]. We have shown that identical TK sequences were recovered from successive CSF samples and samples from distinct anatomical sites (i.e. CSF and BLS from herpetic oral or genital lesions), suggesting that consecutive HSE and mucosal HSV infections within the same individual could be caused by the same isolate. However, given the low genetic variability of HSV-2 TK, sequence analysis of HSV *US4*, *US7* and *US8* genes, commonly used to genotype HSV isolates [45, 46], in addition to TK sequencing in future studies may provide additional insight into the intra-individual HSV-1 and, especially, HSV-2 strain diversity.

In conclusion, we did not detect intrathecal ACV^R^-associated TK mutations in HSV isolates from HSE patients and short-term ACV therapy did not induce emergence of detectable ACV^R^-associated TK mutations. Given the low genetic variability in the TK gene of HSV-2 compared to HSV-1, combined sequence analysis of HSV TK and DNA polymerase genes is needed to conclusively determine the prevalence of ACV^R^ HSV in HSE patients in future studies.
